# A Novel Peptide for Simultaneously Enhanced Treatment of Head and Neck Cancer and Mitigation of Oral Mucositis

**DOI:** 10.1371/journal.pone.0152995

**Published:** 2016-04-06

**Authors:** Peili Chen, Maria Mancini, Stephen T. Sonis, Juan Fernandez-Martinez, Jing Liu, Ezra E. W. Cohen, F. Gary Toback

**Affiliations:** 1 Department of Medicine, University of Chicago, Chicago, Illinois, 60637, United States of America; 2 Biomodels, LLC, Watertown, Massachusetts, 02472, United States of America; 3 Brigham and Women's Hospital, Boston, Massachusetts, 02115, United States of America; 4 Mathematics Department, Universidad de Oviedo, Asturias, Spain; University of Sheffield, UNITED KINGDOM

## Abstract

We have characterized a novel 21 amino acid-peptide derived from Antrum Mucosal Protein (AMP)-18 that mediates growth promotion of cultured normal epithelial cells and mitigates radiation-induced oral mucositis in animal models, while suppressing *in vitro* function of cancer cells. The objective of this study was to evaluate these dual potential therapeutic effects of AMP peptide in a clinically relevant animal model of head and neck cancer (HNC) by simultaneously assessing its effect on tumor growth and radiation-induced oral mucositis in an orthotopic model of HNC. Bioluminescent SCC-25 HNC cells were injected into the anterior tongue and tumors that formed were then subjected to focal radiation treatment. Tumor size was assessed using an *in vivo* imaging system, and the extent of oral mucositis was compared between animals treated with AMP peptide or vehicle (controls). Synergism between AMP peptide and radiation therapy was suggested by the finding that tumors in the AMP peptide/radiation therapy cohort demonstrated inhibited growth vs. radiation therapy-only treated tumors, while AMP peptide-treatment delayed the onset and reduced the severity of radiation therapy-induced oral mucositis. A differential effect on apoptosis appears to be one mechanism by which AMP-18 can stimulate growth and repair of injured mucosal epithelial cells while inhibiting proliferation of HNC cells. RNA microarray analysis identified pathways that are differentially targeted by AMP-18 in HNC vs. nontransformed cells. These observations confirm the notion that normal cells and tumor cells may respond differently to common biological stimuli, and that leveraging this finding in the case of AMP-18 may provide a clinically relevant opportunity.

## Introduction

Despite its frequency, severity and symptomatic and economic impact, there is no effective intervention for oral mucositis (OM) induced by chemotherapeutic or radiation regimens (CRT) used to treat head and neck cancers (HNC) [1–4]. Clinically, OM is characterized by mucosal breakdown resulting in extensive, deep ulcerations, severe function-altering pain, increased risk of secondary infection, bacteremia and sepsis, and expanded need for gastrostomy feedings, and ambulatory and in-patient supportive care. Virtually all patients who receive CRT for the treatment of HNC develop at least moderate OM; more than two-thirds suffer from severe forms of the condition.

Current standard therapy for mucositis is predominantly palliative while focused on pain control and maintenance of nutrition. The only approved treatment for mucositis thus far is palifermin (Kepivance®), and its application is limited to mucositis in patients undergoing conditioning regimens prior to hematopoietic stem cell transplant [5].

The complexity of mucositis as a biological process has only been appreciated recently [6–8]. It has been suggested that the condition represents a sequential interaction of oral mucosal cells and tissues, reactive oxygen species, pro-inflammatory cytokines, mediators of apoptosis, and local factors such as saliva and the oral microbiota. It appears that the early changes associated with radiation-induced mucosal toxicity occur within the endothelium, and connective tissue of the submucosa [6]. This ultimately results in damage and disruption of the epithelial barrier, the most critical step in the development of mucositis. Epithelial barrier function largely depends on the intercellular junctions at the apical-most domains of the plasma membrane, i.e., the tight junctions (TJs) that regulate paracellular permeability across the epithelium in monolayer cell cultures and *in vivo* [9].

We have identified and characterized a novel 21-amino acid peptide derived from amino acids 77–97 of Antrum Mucosal Protein (AMP)-18, also known as gastrokine-1 (GKN1) [10–12], that was initially discovered in epithelial cells of the gastric mucosa [10]. Both AMP-18 and the peptide protect against and speed recovery from mucosal injury in animal models of OM induced by radiation and/or chemotherapy as used to treat patients with HNC [13, 14]. The peptide can be easily synthesized, and exhibits long-term stability. In a mouse model of OM induced by a single dose of radiation to the snout, AMP peptide administered once daily four days before radiation and continued 10 days afterwards effectively prevented the complete loss of tongue epithelium observed in irradiated mice given vehicle (saline) [13]. In established hamster OM models induced by a single dose of radiation, fractionated radiation, or fractionated radiation together with cisplatin to simulate conventional treatments of HNC, daily subcutaneous administration of AMP peptide delayed the onset of mucosal erythema, reduced the severity of ulceration and accelerated oral mucosal recovery in all three models, likely in part by its anti-apoptotic effects seen in cultures of epithelial and endothelial cells [14].

In contrast to other currently trialed or symptom-relieving agents now in use to treat oral mucositis, AMP peptide and recombinant human (rh) AMP-18 appear to uniquely target TJs that connect adjacent epithelial cells by increasing accumulation of TJ proteins, limiting their loss during injury, and facilitating formation of new TJs following mucosal barrier injury [12, 15]. This TJ-enhancing effect is closely associated with rearrangement of perijunctional actin and formation of ‘‘polarity protein” complexes. AMP-18 appears to achieve its therapeutic effects by binding to the gastrin/cholecystokinin B receptor (CCKBR) and activating multiple signaling pathways such as MAPKs, PI3K/AKT, RhoA, and PKCζ [13, 15, 16].

To continue development of AMP peptide as an effective and safe therapeutic agent, its effects on growth of cancer cells in the setting of radiation therapy protocols *in vivo* were studied in a xenograft model using inoculated human carcinoma cells to generate tumors in nude rats [14]. We reported that focal radiation treatment significantly reduced tumor volume as expected. Unexpectedly, administration of AMP peptide significantly added to radiation-induced growth inhibition, demonstrating the tumor suppressor property of AMP-18 we and others observed previously [11, 17, 18]. Together these findings suggested that AMP peptide treatment of OM would not interfere with the therapeutic effects of radiation protocols in the treatment of head and neck tumors, or promote growth of cancer cells, but could instead sensitize tumor cells to radiation.

In this study we set out to determine if AMP-18 could sensitize HNC cells to chemotherapeutic agents, and also demonstrate in the same animal, beneficial effects of oral mucosal protection along with suppression of tumor cell growth following radiotherapy.

## Materials and Methods

### Materials

Chemically synthesized AMP peptide (LDALVKEKKLQGKGPGGPPPK), a scrambled peptide (GKPLGQPGKVPKLDGKEPLAK), and recombinant human (rh)AMP-18 were prepared by GenScript (Piscataway, NJ) as described previously [13]. Briefly, rhAMP-18 is expressed and purified as a His_6_-tagged fusion protein. The coding sequence for full-length human AMP-18 was cloned into an *E*. *coli* expression vector, pGSE3, and the expressed protein was purified from 5 L of culture medium by affinity column chromatography. AMP peptide and rhAMP-18 were found to be equally effective in triggering cellular responses as previously reported [13–15]. Cell culture medium, fetal bovine serum (FBS), penicillin and streptomycin were obtained from Gibco BRL, Life Technologies (Gaithersburg, MD). Tumor necrosis factor (TNF)-α was purchased from PeproTech (Rocky Hill, NJ), and other reagents from Sigma-Aldrich unless otherwise specified.

### Cell Cultures

To investigate the effects of treatment with AMP peptide or AMP-18 in cancer cells exposed to cisplatin, two established human HNC cell lines that were shown in preliminary studies to exhibit differential sensitivity to cisplatin were used: SCC-25 (American Type Culture Collection, ATCC CRL1628) (Manassas, VA) which is more sensitive than SCC-61 [19]. The SCC-25 cell line has been maintained in the authors’ lab for 3 years; SCC-61 cells were established and authenticated by Weichselbaum *et al*. [20–22], received as a gift, and maintained for 4 years. Cells were grown in a 1:1 mixture of Dulbecco's modified Eagle's medium and Ham's F12 medium containing 1.2 g/L sodium bicarbonate, 2.5 mM L-glutamine, 15 mM HEPES and 0.5 mM sodium pyruvate, and supplemented with 400 ng/ml hydrocortisone, 10% FBS, penicillin (50 U/mL) and streptomycin (50 μg/mL), at 37°C in a humidified incubator supplemented with 5% CO_2_. SCC-25 and SCC-61 cells (2 × 10^3^ /well) were grown on 96-well plates for 24 h in medium containing 1% FBS, and were then left untreated, exposed to cisplatin only (2 μM for SCC-25 cells, 10 μM for SCC-61 cells), cisplatin in the presence of 2 μg/ml AMP peptide, or rhAMP-18 (dissolved in phosphate buffered saline, PBS, vehicle) for 48 h. Cell viability was assayed by CellTiter-Blue reagent using a microplate reader (Bio-Tek, Winooski, VT). Experiments were performed in quadruplicate. Human, adult, (low concentration of) Calcium, elevated Temperature (HaCaT) skin keratinocytes, a nontransformed cell line used to model normal stratified squamous epithelium of the oral mucosa [23], were obtained from Thermo Fisher Scientific (Waltham, MA) and maintained in Dulbecco's modified Eagle's medium with FBS, penicillin and streptomycin as described [13] for 5 years. Oral TERT-2 immortalized keratinocytes were considered as a cell model to compare to oral cancer [24], but the HaCaT cell line was chosen because it has been more widely used for this purpose.

### Creation of Orthotopic Model of HNC in Nude Mice

To study possible synergistic effects of AMP peptide on limiting cancer cell growth while protecting against OM in the same animal, an orthotopic model of HNC was used. Tumors in the tongue were generated, their size was assessed using an *in vivo* imaging system (IVIS), and the extent of radiation-induced OM was compared between animals treated with AMP peptide or vehicle (controls). Animal use was approved by Biomodels’ Institutional Animal Care and Use Committee (12-1016-01). The Office of Laboratory Animal Welfare assurance number is A4591-01.

Based on preliminary *in vivo* studies, SCC-25 cells were labeled with a lentiviral dual bioluminescent and fluorescent imaging vector UBC-GFP-T2A-Luciferase (LUC) (System Biosciences, Inc., Mountain View, CA), and injected into the anterior tongue to form tumors. These bioluminescent SCC-25 cells, referred to as SCC-25-LUC, enable *in vitro* selection of positive cells by flow cytometric sorting using green fluorescent protein (GFP) and non-invasive monitoring of cellular dynamics utilizing an *in vivo* imaging system (IVIS) (Perkin Elmer) to measure tumor size. SCC-25-LUC cells were sorted 3 times by BD FACSAria (BD Biosciences) so that GFP-positive cells were >95% (data not shown). The sensitivity and accuracy of *in vitro* and *in vivo* cell monitoring offers several advantages over traditional methods that require sacrificing animals and histological analysis. In addition, histopathological correlates of the clinical changes assessed in the model have been extensively documented [13, 25], so that histology was not a part of the current study.

Athymic nude mice (Foxn1^nu^) (age 6 to 8 weeks, body weight 29.5 ± 2.0 g) were purchased from Charles River Laboratories. Mice were anesthetized with isoflurane and 1 x 10^6^ SCC-25-LUC cells suspended in 30 μL 1x HBSS were injected directly into the anterior tongue using a 0.1 mL tuberculin syringe with a 30 gauge needle, as described by Myers *et al*. [26]. After 21 days to permit tumor growth, a total of 32 mice were randomized into 4 groups (8 mice/group) based on a quantitative assessment of tumor size as determined by total flux from each tumor measured by IVIS imaging [27].

The day of randomization was designated Day 0. Animals in Group 1 were not treated, Group 2 mice received vehicle (PBS), and Groups 3 and 4 received AMP peptide at a dose of 25 mg/kg once daily via subcutaneous (s.c.) injection. On study Day 4, animals in Groups 2 and 4 were given a single acute dose of 30 Gy of radiation directed to the tongue to treat the tumors and induce OM. The model isolates the target tissue (tongue) with a lead shield, thereby avoiding systemic toxicity. Following administration of anesthesia, mice were radiated with using a 160 kilovolt potential (18.75-ma) source at a focal distance of 15 cm, hardened with a 3.0 mm Al filtration system. Treatment with AMP peptide or vehicle was continued for 14 days.

Animals were anesthetized, their tongues gently extruded using a forceps and photographs were obtained to document tumor progression. Tumor growth was assessed and compared on Days 5, 8, 10 and 14 using IVIS after injection of D-luciferin. After photographs were taken, OM was scored clinically, and animals were imaged using the IVIS system. Prior to imaging, mice were injected with 0.1 mL / 20 g body weight of 15 mg/mL D-luciferin-K^+^ bioluminescent substrate in PBS. Ten minutes following injection, mice were anesthetized and placed in the IVIS® Lumina at maximum sensitivity for up to 5 minutes exposure to detect bioluminescence with an open emission filter. Saved images were loaded into Living Image® analysis software and color scales were matched based on maximum and minimum radiance (photons/second/cm^2^/steradian). Identical regions of interest were drawn for each animal and total flux was determined in terms of photons/second for each region of interest.

### Oral Mucositis

**Fig 1:**
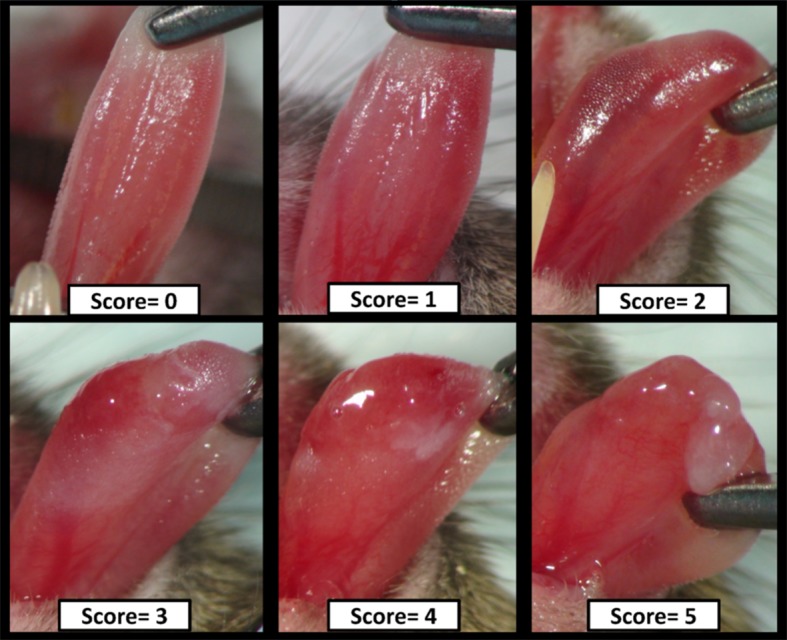
Mucositis Scoring Scale. Representative images of radiation-induced mucositis used for scoring are shown. See details in the Methods.

The onset and progression of OM was scored clinically using a well-established six point scale: Grade 0, no abnormalities. Grade 1, slight mucosal changes characterized by areas of mild erythema, mucosa intact. Grade 2, mild mucositis defined by areas of moderate to severe erythema and superficial epithelial necrosis such that there may be mild sloughing, but the epithelial base is intact. Grade 3, moderate mucositis characterized by frank ulcer formation. Ulcers typically have areas of necrosis with associated yellowish/gray coloration with pseudomembrane formation. Cumulative area of ulceration ≤ 25% of tongue surface area. Grade 4, severe mucositis with ulcer formation affecting 25% to 50% of tongue mucosa. Marked erythema and pseudomembrane formation. Loss of pliability. Grade 5, severe mucositis ulcer formation of virtually all mucosa areas at risk. Loss of mobility and pliability [25, 28, 29]. Scores of 1 and 2 indicate mild stages of mucositis. Ulcerative mucositis is characteristic of grades 3–5 with grades 3 and 4 representing severe stages of the condition. Representative images of this scale are defined in Fig 1. Following visual clinical scoring, a photograph was taken of each animal’s oral mucosa using a standardized technique. At the conclusion of the experiment, photographs were randomly numbered and scored by two independent, trained observers who graded the images in blinded fashion using the above-described scale (blinded scoring). For each photograph, the actual blinded score was based on the average of the 2 observers’ scores. Only scores from the blinded, photographic evaluation were reported and used for statistical analyses.

Animals were weighed and monitored for general health daily. In addition to the standard diet, all animals were given highly palatable soft food in their cages. Group weight change was recorded as a mean percent weight change.

### Sodium Dodecyl Sulfate-Polyacrylamide Gel Electrophoresis (SDS-PAGE) and Immunoblotting Assay of Caspase 3 Cleavage

To determine the role of apoptosis as a conduit for disparate AMP-18 effects on normal epithelial and HNC cancer cells, caspase 3 cleavage was used as an index of cell apoptosis and compared in nontransformed HaCaT and HNC SCC-25 cells exposed to TNF-α using immunoblotting. To prepare cell lysates, cultures were rinsed and then harvested in iced PBS by scraping the monolayer with a cell lifter. The detached cells were pelleted at 4°C and extracted on ice for 30 min in lysis buffer (50 mM Tris-HCl, pH 7.4, 1% Nonidet P-40, 0.25% sodium deoxycholate, 150 mM NaCl, 100 mM NaF, 10% glycerol, 10 mM EDTA) containing protease and phosphatase inhibitors (2 mM sodium orthovanadate, 1 mM phenylmethylsulfonyl fluoride, 50 μg/mL antipain, 1 μg/mL aprotinin, 1 μg/mL leupeptin, and 1 μg/mL pepstatin). Cell lysates were clarified by centrifugation at 14000 × *g* for 15 min at 4°C. Protein concentration was determined by BCA assay (Pierce).

For immunoblotting assays, 30 to 50 μg protein/lane was resolved by SDS-PAGE, transferred onto Immobilon membranes (Millipore, Bedford, MA) followed by blocking with 5% bovine serum albumin in TBST (20 mM Tris-HCl, 150 mM NaCl, 0.1% Tween 20 at pH 7.5), and incubated with designated antibodies. After incubation with horseradish peroxidase (HRP)-conjugated secondary antibodies, immunoreactive bands were visualized using chemiluminescence (ECL, Amersham Biosciences). When reprobed, blots were first stripped with a buffer containing 50 mM Tris-HCl, pH 6.8, 2% SDS, and 0.1 M 2-mercaptoethanol. Images were analyzed by densitometry. The immunoblots shown in the figure represent one of at least three experiments.

### RNA Microarray Analysis Comparing Nontransformed and HNC Cells Treated with AMP-18

To define the differential biological impact and relevance of AMP-18 on normal and tumor tissue, and to identify putative pathway gene targets that could mediate the dual therapeutic effects we compared its effect on gene expression of cultured nontransformed (HaCaT) or squamous cell carcinoma cells treated with AMP-18.

We [13, 14] and others [30–34] have utilized the human immortalized nontransformed skin keratinocyte HaCaT line [23] to model the oral mucosal epithelium and for comparison with squamous head and neck cancer cells, whereas immortalized oral TERT-2 keratinocytes have been used less often to model oral mucositis.

Total RNA was extracted from SCC-61 and HaCaT cells, with or without AMP-18 treatment, using the RNeasy kit (Quiagen) according to the manufacturer’s specifications. RNA integrity and concentration were assessed using an Agilent Bioanalyzer 2100. Total RNA was processed into biotinylated cRNA using the Illumina® TotalPrep™-96 RNA Amplification Kit (Ambion, cat no: 4393543). The cRNA was hybridized to Illumina HumanHT12v4 arrays using Illumina provided protocols and scanned using an Illumina HiScan in the University of Chicago Genomics Core laboratories.

Using microarray data from HaCaT and SCC-61 cells obtained at 2 hours and 24 hours after exposure to AMP-18 (and no treatment controls), we used an approach that optimized an initial supervised component with subsequent statistically driven hierarchical ranking. Genes which were most discriminatory between the two groups were ranked according to their discriminatory value as defined by their Fisher’s ratio in which the predictive accuracy of the different ordered reduced sets were determined using a backward recursive feature elimination algorithm. This procedure eliminated redundant or irrelevant genes to yield the most precise set of genes with the greatest predictive value. We then evaluated the most differentially expressed genes for disease attribution, pathway association, and gene ontology using GeneAnalytics software and tested functional/phenotypic associations using VarElect [8, 35, 36]. The top 20 genes differentially expressed in SCC-61 and HaCaT cells treated with AMP-18 for 2 h are shown in S1 Table.

### Statistical Analyses

Mucositis was scored using the blinded photographs, beginning on Day 4, and for every day thereafter. Statistical differences between treatment groups were determined using the Mann-Whitney Rank Sum test and chi-square analysis with a critical value of 0.05. The effect of drug treatment on mucositis compared to vehicle control was assessed according to the following parameters: 1. The difference in the number of days mice in each group had severe mucositis (score ≥ 3) was analyzed. On each day the animals were scored (evaluation day), the number of animals with a blinded mucositis score of ≥ 3 in each treatment group was compared to the vehicle group. Differences were analyzed on a daily as well as a cumulative basis. Treatment success was determined by a statistically significant lower number of mice with this score in a drug treatment group, versus vehicle, as determined by chi-square analysis. 2. The rank sum differences in daily mucositis scores in treatment groups versus the vehicle group were determined. For each evaluation day the scores of the vehicle group were compared to that of the treated groups using non-parametric rank sum analysis. Treatment success was determined by a statistically significant lowering of scores in the treated group on two or more days from Day 4 to Day 14. A one-way ANOVA or ANOVA on ranks was used to evaluate the area-under the curve for animal weights and IVIS measurements. Data were analyzed with Minitab software (Minitab, State College, PA). Groups were compared by two-tailed *t* test or, for more than two groups, by analysis of variance. We considered *P* ≤ 0.05 significant.

## Results

### Inhibitory Effects of AMP Peptide and rhAMP-18 on HNC Cells

**Fig 2:**
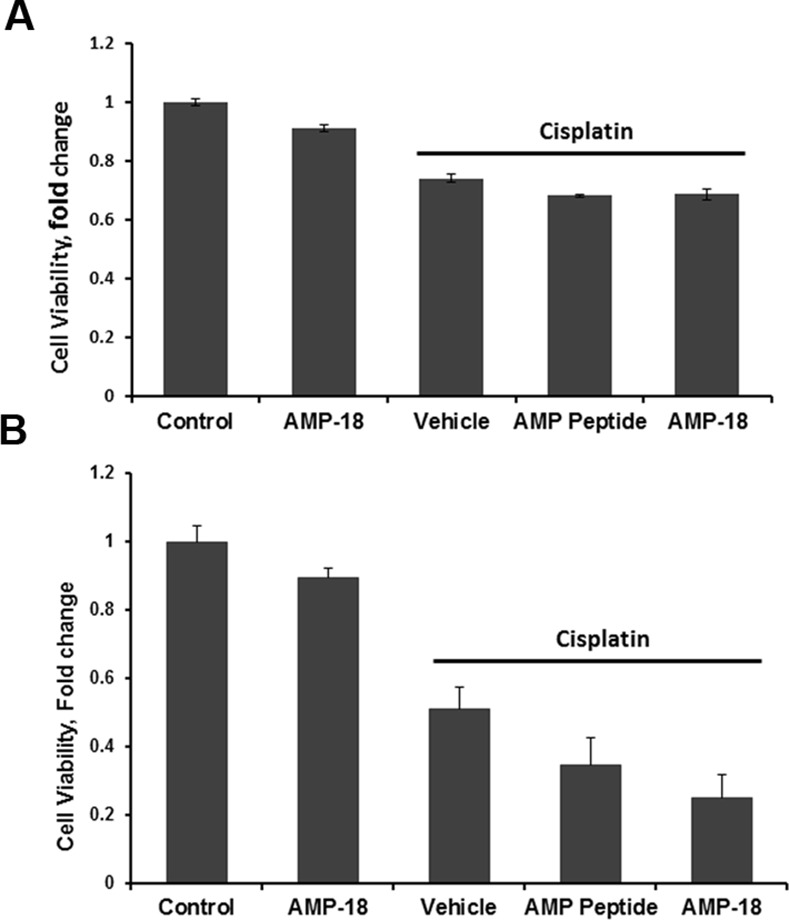
Inhibitory effects of AMP peptide and AMP-18 on HNC cells. SCC-25 (**A**) or SCC-61 (**B**) cells were left untreated, exposed to AMP-18 (2 μg/ml), cisplatin (2 μM for SCC-25 cells, 10 μM for SCC-61 cells) only, cisplatin in the presence of 2 μg/ml AMP peptide, or rhAMP-18 for 48 h. Cell viability was assayed by CellTiter-Blue reagent. Experiments were performed in quadruplicate. Values are mean ± SE. Adding either AMP peptide or rhAMP-18 amplified the cytotoxicity of cisplatin by inhibiting growth of SCC-25 cells by >30% (AMP peptide, P = 0.013; rhAMP-18, P = 0.03), and SCC-61 cells by 65% (P<0.05) and 75% (P<0.02), respectively.

To investigate the effects of AMP peptide or AMP-18 on HNC cells in culture, human head and neck squamous cell carcinoma SCC-25 (Fig 2, panel A) and SCC-61 (panel B) cells were left untreated, exposed to AMP-18 (2 μg/ml), cisplatin (2 μM for SCC-25 cells, 10 μM for SCC-61 cells) only, cisplatin in the presence or absence of AMP peptide or rhAMP-18. Cell viability was assessed with the CellTiter-Blue® Cell Viability Assay reagents (Promega) using a microplate reader. AMP-18 alone inhibited growth of SCC-25 (P = 0.02) and SCC-61 (P = 0.04) cells by 10–15% (Fig 2). Cisplatin significantly inhibited growth of both cell lines: SCC-25 cells by 25%, and SCC-61 cells by 50% compared to control cultures (P<0.001). Adding either AMP peptide or rhAMP-18 amplified the cytotoxicity of cisplatin by inhibiting growth of SCC-25 cells by >30% (AMP peptide, P = 0.013; rhAMP-18, P = 0.03), and SCC-61 cells by 65% (P<0.05) and 75% (P<0.02), respectively.

### AMP Peptide Treatment Additively Inhibits Cancer Growth with Radiation

Previous evidence that AMP peptide could sensitize cancer cells to radiation in a xenograft model [14] and two human HNC cell lines to cisplatin in culture (Fig 2), together with our studies in mice and hamsters that treatment with the peptide had beneficial clinical effects in radiation- and cisplatin-induced OM [13, 14], led us to ask if these dual effects of AMP peptide could be demonstrated in an orthotopic model of HNC in the same animal. Four groups of animals were studied: 1. untreated; 2. PBS (vehicle) and radiation; 3. AMP peptide; and 4. AMP peptide and radiation.

**Fig 3:**
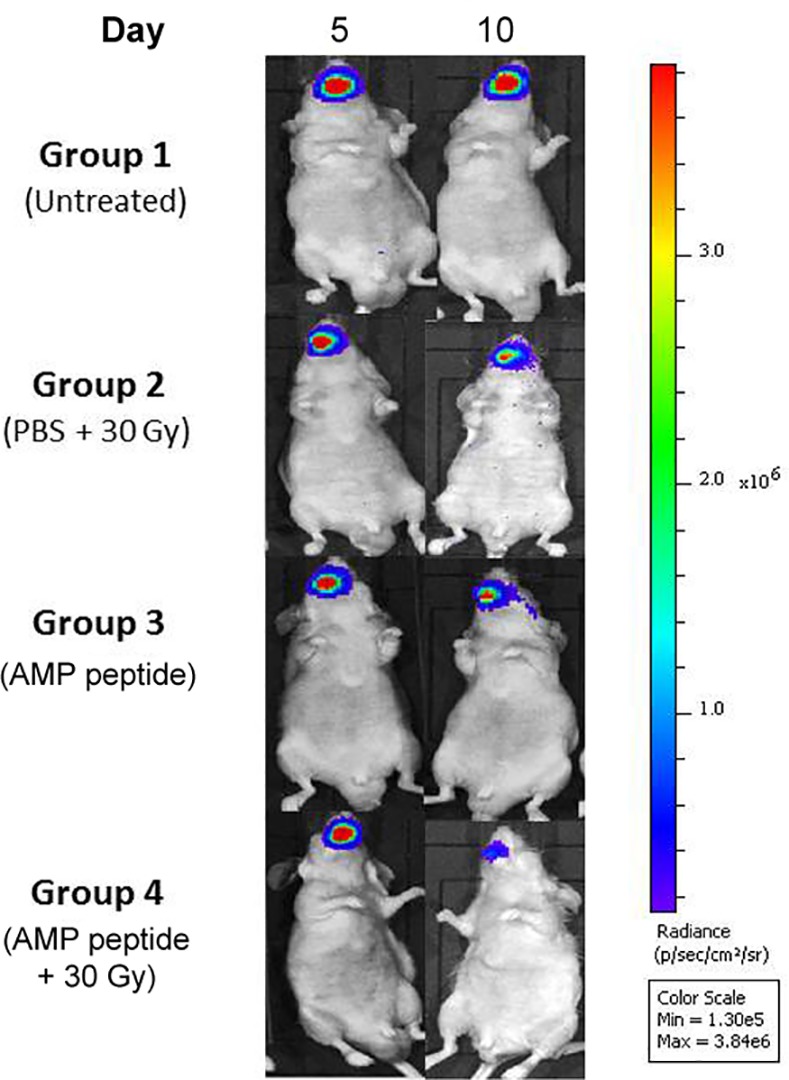
Effect of AMP peptide and radiation treatment in an orthotopic model of HNC cancer in the tongue assessed using IVIS. An orthotopic model of HNC cancer was established by inoculating bioluminescent SCC-25-LUC cells into the anterior tongue of nude mice. Tumor size in different groups was assessed and compared on Days 5 and 8 as well as Days 10 and 14 using IVIS after injection of D-luciferin. Mice in Group 1 and Group 3 were not irradiated, whereas animals in Groups 2 and 4 received 30 Gy on Day 0 of the study. Treatment with PBS (Group 2) or AMP peptide (Group 4) was administered by injection s. c. once daily. In the irradiated mice, treatment with AMP peptide was associated with much lower IVIS radiance compared to treatment with PBS, indicating that AMP peptide reduced tumor growth. Representative IVIS images of tongue tumors from 32 mice are shown.

**Fig 4:**
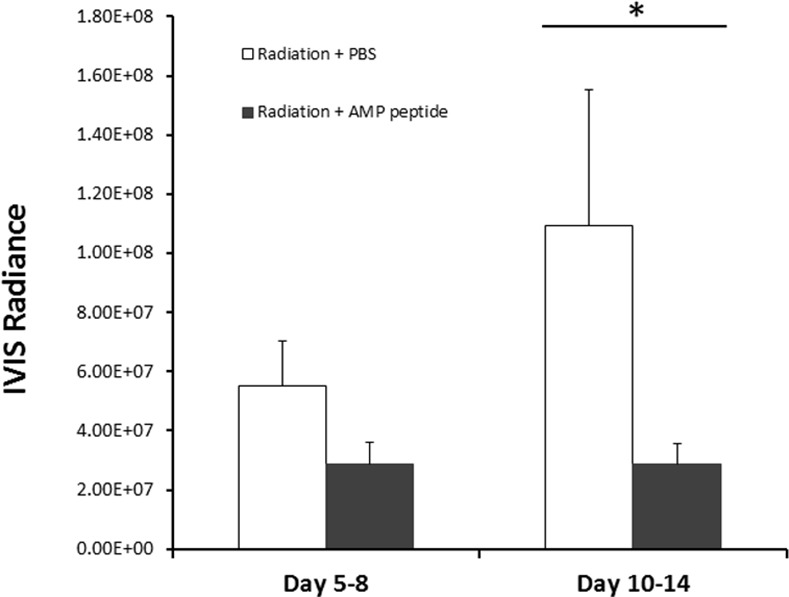
Effect of AMP peptide and radiation treatment of HNC tumors in the anterior tongue of nude mice with time assessed by measuring tumor IVIS radiance. Irradiated animals were treated with AMP peptide or PBS, and IVIS radiance was measured on Days 5 and 8, and then on Days 10 and 14. Means ± SE were compared. In mice given PBS, IVIS radiance appeared to increase between Days 5 and 8 and 10 and 14, whereas radiance in animals treated with AMP peptide was unchanged between Days 5 to 14. On Days 10 and 14, treatment with AMP peptide significantly reduced tumor size compared to treatment with PBS (* P = 0.03). In non-irradiated mice, the orthotopic tumors in the tongue grew progressively; administration of AMP peptide did not change tumor size significantly (not shown).

To determine the impact of treatment with AMP peptide on tumor progression with and without radiation therapy, the tumors of all animals were assessed using the IVIS system. In non-irradiated mice, the orthotopic tumors in the tongue grew progressively, and administration of AMP peptide did not change tumor size significantly (Groups 1 and 3) (Fig 3). On Days 5 and 8 IVIS values for untreated (6.59×10^7^ ± 9.6×10^6^) and AMP peptide treated (6.50×10^7^ ± 8.67×10^6^) were not statistically different, as was also the case on Days 10 and 14 when IVIS values for untreated (1.78×10^8^ ± 2.47×10^7^) and AMP peptide treated (1.61×10^8^ ± 2.32×10^7^) did not differ. In mice given radiation (30 Gy), tumor size, measured as mean IVIS radiance, continued to grow with PBS (vehicle) treatment (Group 2), but remained unchanged with AMP peptide treatment between Days 5 and 8 and Days 10 and 14 (Group 4) (Fig 4). Tumor size (means ± SE) in radiated mice, given AMP peptide compared to mice given PBS, was significantly reduced (P = 0.03) when assessed on Days 10 and 14 (Fig 4). This observation suggested that AMP peptide did not interfere with the ability of focused radiation to inhibit cell growth, but rather halted growth of cancer cells.

AMP peptide did not exhibit any adverse effects on body weight. Mice in all 4 groups exhibited weight loss after tumors were established in the tongue which was more marked in irradiated animals (data not shown).

### Therapeutic Effects of AMP Peptide on Radiation-Induced Oral Mucositis

**Fig 5:**
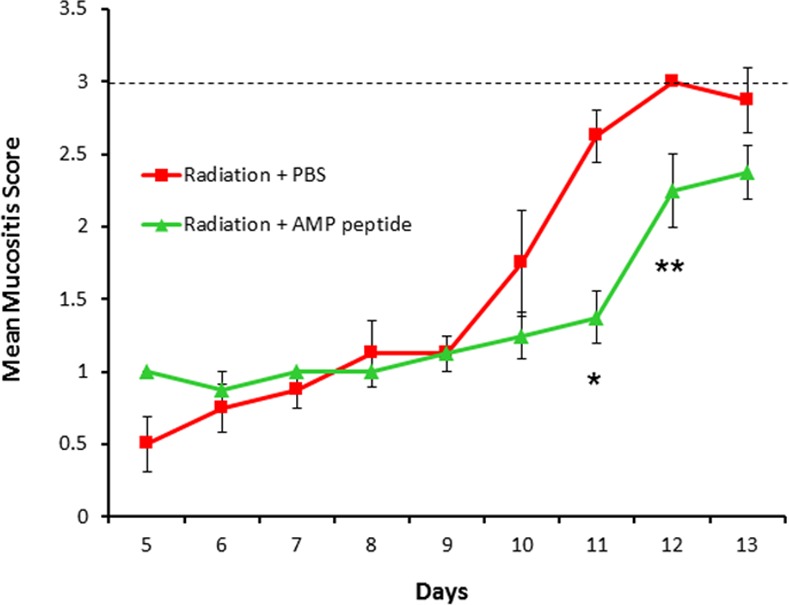
Mean daily mucositis scores in irradiated mice. Irradiation of the tongue and oral mucosa was followed by daily treatment with AMP peptide or vehicle (PBS). Ulcerative oral mucositis that developed was scored as described in *Methods*. A mucositis score of 3.0 (broken line) indicates mucosal ulceration that correlates with significant human oral mucositis. Animals treated with the vehicle reached a score of 3 on Day 12, whereas mice treated with AMP peptide did not. Mucositis score was significantly lower on Days 11 (* P = 0.003) and 12 (** P = 0.03), but not Day 13 (P = 0.1) in animals treated with AMP peptide compared to those given the vehicle. Values are means ± SE.

In agreement with the results of previous studies, AMP peptide mitigated the development oral mucositis induced by radiation [14]. While radiated control (PBS) animals all developed ulcerative mucositis (Group 2) by day 12, only 37.5% of AMP-treated animals (Group 4) were so affected (P = 0.03), whereas on day 11, mucosal ulceration appeared in 62.5% in Group 2, but none given AMP peptide (P = 0.003) (Fig 5). Thus, AMP peptide delayed the onset of ulcerative mucositis and favorably impacted its duration.

### AMP-18 Inhibited Apoptosis in HaCaT but Not in HNC Cells

Recent results from our studies [10–15] and others [17] suggest that AMP-18 has pleiotropic effects. AMP-18 stimulates growth and is anti-apoptotic in epithelial cells including HaCaT keratinocytes [13]. It also appears to function as a tumor suppressor [37], possibly by inducing apoptosis [38], inhibiting the epithelial–mesenchymal transition (EMT) and cancer cell migration [39], and/or inducing cellular senescence [40]. Indeed, expression of AMP-18 is downregulated or absent in gastric cancer tissue [11, 17, 18], and decreases as chronic gastritis progresses to atrophy and metaplasia [40, 41].

To determine if apoptosis could be a mechanism by which AMP-18 exerts its dual effects on normal epithelial and HNC cancer cells observed in the orthotopic model, the effect of AMP-18 on caspase 3 cleavage as an index of cell apoptosis was compared in nontransformed HaCaT and HNC SCC-25 cells exposed to TNF-α, a proinflammatory cytokine known to mediate chemo- and radiation-therapy induced injury of the oral mucosa.

**Fig 6:**
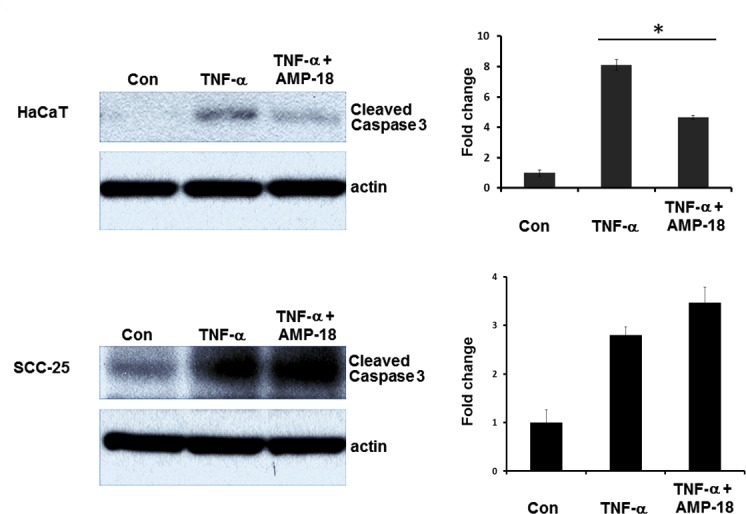
Differential effects of AMP-18 on cleavage of caspase 3 in HaCaT and SCC-25 cells. Nontransformed human keratinocyte (HaCaT cells) and human HNC (SCC-25) cell cultures were each exposed to TNF-α for 6 h to induce apoptosis displayed as increased cleavage of caspase 3; 8.1-fold in HaCaT, and 2.8-fold in SCC-25 cells. Treatment with rhAMP-18 appeared to block TNF-α –induced cleavage of caspase 3 in HaCaT (50–75%, * P = 0.02) but not in HNC cells. Each immunoblot represents one of three experiments.

Minimal apoptosis was observed in both cell lines under control conditions whereas exposure to TNF-α induced a significant increase in cleaved caspase 3 (Fig 6); 8.1-fold in HaCaT, and 2.8-fold in SCC-25 cells. However, in HaCaT cells treated with AMP-18, caspase 3 cleavage was inhibited by 50–75% (P = 0.02), suggesting an anti-apoptotic effect of AMP-18. In contrast, treatment of SCC-25 cells with AMP-18 did not inhibit TNF-α induced cleavage of caspase 3 in SCC-25 cells, indicating a different response in these cancer cells.

### Pathways differentially targeted by AMP-18 in SCC-61 and HaCaT cells

As SCC-61 cells were found to be more sensitive than SCC-25 cells to the additive inhibitory effects of AMP-18/peptide with cisplatin on cell viability (Fig 2), SCC-61 cells were chosen for microarray analysis. Consistent with the different effects of AMP-18 noted in the tumor modeling component of the study (Figs 4 and 5), a differential impact of AMP-18 on HaCaT (nontransformed immortalized skin keratinocytes) vs. SCC-61 (HNC) cells was also seen using microarray analysis (GEO number: GSE73330).

As expected, assessment of baseline gene expression of SCC-61 cells identified genes associated with head and neck squamous cell carcinoma and tongue squamous cell carcinoma. Analysis of pathways associated with AMP-18 treatment (at 2 hours) of SCC-61 cells vs. AMP-naïve SCC-61 cells, suggested that AMP-18 initiated differential gene expression associated with pathways functionally related to cell metabolism, oxidation, and tumor behavior (Table 1, *left column*).

**Table 1:** Hierarchically arranged lists of pathways differentially targeted by AMP-18 in SCC-61 and HaCaT cells. SCC-61 and HaCaT cells were treated with AMP-18 for 2 h, total RNA was purified and subjected to RNA microarray analysis. Differentially expressed pathways were identified as stated in the Methods with no AMP-18 treatment as a control.

SCC-61 cells	HaCaT cells	HaCaT vs. SCC-61 cells
• Fatty acid biosynthesis	• microRNAs in cardiomyocyte hypertrophy	• Selenium pathway
• Validated targets of C-MYC transcriptional repression	• (S)-reticuline biosynthesis II	• Immune response IFNα/β signaling pathway
	• Physiological and pathological hypertrophy of the heart	
• Fatty acid metabolism	• Glypican 2 network	• Cell adhesion-ECM remodeling
• Transport of glucose and other sugars, bile salts and organic acids, metal ions and amine compounds	• Glycine cleavage	• Collagen biosynthesis and modifying enzymes
• P70S6K signaling	• Pyrimidine ribonucleosides salvage I	• Degradation of extracellular matrix
• Mitochondrial LC-Fatty acid beta-oxidation	• Guidance cues and growth cone motility	• Metabolism of O-linked glycosylation of mucins
• Platelet homeostasis	• Netrin signaling	• Complement and coagulation cascades
• LDL oxidation in atherogenesis	• Basal cell carcinoma	• Interferon signaling
• MSP-RON signaling	• Defensins	• Cytoskeleton remodeling neurofilaments
• Arf1 Pathway	• Sweet taste signaling	• HIF-1 alpha transcription factor network
• Lamivudine Pathway, Pharmacokinetics/Pharmacodynamics	• Antiarrhythmic pathway	• PAK pathway
• Acetaminphen Pathway, Pharmacokinetics		• Non-integrin membrane-ECM interactions
• Nitric Oxide Stimulates Guanylate Cyclase		• Dissolution of fibrin clot
• AKT Signaling		• All-trans-retinoic acid mediated apoptosis
• Immune Response-MIF-mediated glucocorticoid regulation		• Formation of fibrin clot (clotting cascade)
• Doxorubicin Pathway, Pharmacokinetics		• Matrix metalloproteinases
• CREB Pathway		• Legionellosis
• Axon Guidance		• Validated transcriptional targets of AP1 family members Fra1 and Fra2
• pERK Regulated gene expression		• Rheumatoid arthritis
		• ERK signaling

HaCaT cells exposed to AMP-18 for 2 hours resulted in an entirely different set of expressed genes, as indicated by their functional relationships (Table 1, *middle column*). To better understand the differential effects of AMP-18 on normal (HaCaT) and tumor (SCC-61) cells, we assessed the functional significance of differentially expressed genes after a 2 hour exposure to AMP-18. In contrast to the pathways noted when only SCC-61 cell gene expression was studied, we noted a high prevalence of functions associated with pathways typically correlated with tumor behavior. In particular, a number of pathways identified are strongly associated with local tumor spread and invasion characteristics (Table 1, *right column*).

## Discussion

We have characterized a novel peptide derived from Antrum Mucosal Protein (AMP)-18 that demonstrates pleiotropic effects including growth promotion, anti-apoptosis, and tight junction (TJ) enforcement in animal models of OM [13, 14]. In a xenograft model reported previously, AMP peptide sensitized human cancer cells to radiation therapy. In the present study, we have extended this observation to cultured human HNC cells exposed to cisplatin and treated with AMP peptide or full length AMP-18 protein (Fig 2). We also demonstrated in an orthotopic mouse model of squamous cell cancer of the tongue that AMP peptide administered together with radiation exerts both its radioprotective effects on the oral mucosa and tumor-suppressing properties in the same animal (Figs 3–5). Differential effects of AMP-18 on apoptosis in nontransformed compared to cancer cells suggest an underlying mechanism for the dual effects (Fig 6). Consistent with our *in vivo* observations, we showed that AMP-18 differentially impacted normal and HNC cell gene expression. Pathway attribution associated with these differences was also consistent with phenotype. These results provide an experimental rationale to develop AMP peptide as a therapeutic agent to protect against and speed healing in patients with HNC who acquire chemotherapy and/or radiation-induced OM.

Epithelial cells comprise the most important layer of the mucosal barrier. Degeneration and breakdown of the epithelium results in mucosal ulceration. Epithelial barrier function is regulated by the TJs, a multiple protein complex linked to the cytoskeleton that regulates mucosal permeability in both physiological and pathological states [42–44]. The importance of TJs in the pathoetiology of regimen-related mucosal injury is becoming increasingly clear [45]. Thus targeting epithelial cells and TJs to treat OM seems a promising strategy. We have characterized a novel peptide derived from AMP-18 that demonstrates pleiotropic effects that appear to function differently than do other currently trialed or symptom-relieving agents. It is mitogenic, anti-apoptotic and can stimulate formation of TJs in nontransformed epithelia, while also possibly presensitizing transformed epithelial cells of head and neck cancers to apoptosis (Fig 6). With these multiple roles, AMP-18 appears to maintain mucosal barrier function and structure by adjusting cellular homeostatic balance between proliferation and apoptosis [46] and regulating dynamics of TJs. The potent mucosal protective and healing effects of AMP peptide have been observed in our previous studies using established OM models in mice and hamsters [13, 14].

However, treatment with AMP peptide would be untenable if it caused growth of tumor cells or inhibited the antineoplastic effects of radiation or chemotherapy. This concern was proven to be untrue in a xenograft model [14]. Instead, subcutaneous administration of AMP peptide significantly added to radiation-induced inhibition of cancer cell growth, demonstrating tumor suppressor property as we and others have observed [11, 17, 18]. Here we have extended this observation to include human squamous carcinoma cell lines, SCC-25 and SCC-61 treated with cisplatin (Fig 2), and an orthotopic mouse model of HNC. We found that AMP peptide appeared to work synergistically with radiation to favorably impact the course of tumor growth. In contrast animals given vehicle exhibited progressive cancer growth.

In addition to having anti-tumor activity, there was dramatic improvement in the development and progression of OM in the irradiated head and neck tumor bearing animals. Fewer animals treated with AMP peptide developed oral mucosal ulcers in addition to a significant decrease in mucositis scores compared to mice given the vehicle. Further, the onset of mucositis was delayed in AMP peptide treated animals and the peak disease period was shorter (Fig 5). Taken together in this dual animal model of HNC and OM, AMP peptide can protect and speed healing of the injured oral mucosa following radiation therapy, yet additively with radiation inhibit tumor cell growth. As noted above, the stabilizing impact of AMP peptide on TJs might, at least partially account for this observation. In addition the observation that AMP peptide inhibition of TNF-α induced cleavage of caspase 3 in nontransformed HaCaT cells, but not HNC SCC-25 cells might reflect its differential effect on apoptosis. Finally AMP-18 could inhibit tumor growth by inducing cancer cell senescence [47, 48].

AMP-18 impacted the differential expression of a range of genes in SCC-61 cells compared to SCC-AMP-naïve cells and compared to the effect of the peptide on normal HaCaT cells. The most informative data associated with these genomic differences was associated with function and, particularly, their attribution to specific pathways (Table 1). Consequently, the differential gene expression evoked by AMP-18 between HaCaT and SCC cells was of interest. Not surprisingly, given the phenotypic observations noted with AMP-18, its addition for 2 hours to SCC-61 cells activated multiple pathways known to be associated with tumor behavior. Examples of these pathways include fatty acid biosynthesis and metabolism [49], cell proliferation and invasiveness of squamous cell carcinomas of the head and neck [49], the Arf1 [50] and mTOR/P70S6K pathways [51].

Interestingly, pathways associated with the extracellular matrix (ECM) were highly visible among AMP-18 exposed cells. Given the importance of ECM in tumor invasiveness and its specific relationship with epithelium, this finding may have importance in AMP-18’s ability to modify tumor responsiveness, and is consistent with the report of Liu *et al*. [52] in which the authors evaluated an integrated dataset of 4 public oral squamous cell carcinoma datasets and found that alterations in ECM receptor pathways were the most common seen. It is not surprising, therefore, that pathways associated with collagen biosynthesis and matrix metalloproteinases were among those highly ranked in our analysis. Taken together with the observations of the potential of AMP-18 to impact the tumor environment, it could be that the peptide’s effect of tumor behavior is more focused on invasiveness and local and systemic expansion than on direct viability, such as PAK pathway mediated-cell motility.

Finally, the selenium pathway was prominently identified. Its significance in our model has yet to be defined, but enhanced selenoprotein production might be associated with favorable anti-tumor effects, as its depletion has been noted to have the opposite effect [53]. Alternatively, given consistent reports of its ability to enhance tumor cell apoptosis [54], one might hypothesize that AMP-18-mediated stimulation of the selenium pathway resulted in increased levels of metabolites which inhibited observed tumor growth.

In summary, we have identified a novel agent that acts additively with radiation or chemotherapy to limit tumor cell growth while maintaining integrity of oral mucosal barrier function, possibly through proliferative and anti-apoptotic effects in epithelial cells. The dual effects of the peptide have yet to be fully defined, but the data presented here suggest its potential efficacy as a new agent to treat patients with HNC who frequently develop mucositis.

## Supporting Information

S1 Table Top 20 genes with fold changes in SCC-61 and HaCaT cells treated with AMP-18. (PDF)
