# Supplementary material for: A Novel Peptide for Simultaneously Enhanced Treatment of Head and Neck Cancer and Mitigation of Oral Mucositis
Source: PLoS One. 2016 Apr 6;11(4):e0152995. doi: 10.1371/journal.pone.0152995 (PMC4822960; doi:10.1371/journal.pone.0152995)
Supplement: S1 Table — (PDF) [file pone.0152995.s001.pdf]

S1 Table. Top 20 genes with fold changes in SCC-61 and HaCaT cells treated with AMP-18. Cells were exposed to AMP-18 for 2 h, total RNA was purified and subjected to RNA microarray analysis. Differentially expressed genes were identified as stated in the Methods with no AMP-18 treatment as a control.

Gene symbol	Fold change
IL6	31.25
LCN2	28.23
SAA1	18.12
LOC100134134	15.79
TIMP2	14.76
PLAT	14.24
CFB	11.44
SLC16A2	9.63
TTYH3	6.19
TM4SF19	5.78
CEACAM6	5.63
LGALS3BP	5.22
FBLN1	4.91
FAT1	4.56
NEU1	3.97
KRT16	3.97
FAS	3.94
FAM46A	3.87
FAM20C	3.70
